# Tandem mass tagged dataset used to characterize muscle-specific proteome changes in beef during early postmortem period

**DOI:** 10.1016/j.dib.2020.106064

**Published:** 2020-07-23

**Authors:** Chaoyu Zhai, Blanchefort A. Djimsa, Kitty Brown, Jessica E. Prenni, Dale R. Woerner, Keith E. Belk, Mahesh N. Nair

**Affiliations:** a Department of Animal Sciences, Colorado State University, United States; b Department of Animal and Food Sciences, Texas Tech University, United States; c Proteomics and Metabolomics Facility, Colorado State University, United States; d Department of Horticulture and Landscape Architecture, Colorado State University, United States

**Keywords:** Muscle-specificity, TMT, Muscle-to-meat conversion, Meat quality

## Abstract

Bovine *longissimus lumborum* (LL) and *psoas major* (PM) muscles biopsy samples were collected from four carcasses (*n* = 4) at 45 min, 12 h, and 36 h postmortem from a commercial beef processing facility. Proteins present in the early postmortem LL and PM proteomes were identified and quantified using tandem mass tag (TMT) labelled, fractionated peptides coupled to liquid chromatography tandem mass spectrometry (LC-MS/MS). The data are supplied in this article and are related to “Tandem mass tag labeling to characterize muscle-specific proteome changes in beef during early postmortem period” by Zhai et al. [1].

Specifications Table

**Table:** 

**Subject**	Animal Science and Zoology
**Specific subject area**	Meat science, bovine muscle proteome
**Type of data**	Raw data Tables
**How data were acquired**	MS/MS data acquired from mass spectrometer (Orbitrap Velos Pro, Thermo Fisher Scientific) combined with a Nanospray Flex ion source (Thermo Scientific). Compound lists of the resulting spectra were generated using Xcalibur 3.0 software (Thermo Scientific). MS/MS spectra were extracted, charge state deconvoluted and deisotoped by ProteoWizard MsConvert (version 3.0). Spectra from all samples were searched using Mascot (Matrix Science, London, UK; version 2.6.0) against a *Bos taurus* reference proteome downloaded on February 5, 2018 from Uniprot (UP000009136) and reverse concatenated (48,676 total entries). Scaffold software (version Scaffold_4.8.4, Proteome Software Inc., Portland, OR) was used for post-processing and analysis of protein identifications.
**Data format**	Raw, analyzed, and filtered
**Parameters for data collection**	Analysis of protein profile from bovine *longissimus lumborum* (LL) and *psoas major* (PM) muscles at early postmortem timepoints (45 min, 12 h, and 36 h).
**Description of data collection**	Samples originated from 4 animals, 2 tissue types (LL & PM) at 3 timepoints postmortem. Approximately 200 mg of 24 beef samples were submitted for trypsin digest, Tandem Mass Tag (TMT) 10plex labeling, peptide fractionation and protein identification & quantitation via LC-MS/MS.
**Data source location**	Institution: Proteomics and Metabolomics Facility, Colorado State University City: Fort Collins, Colorado Country: United States GPS coordinates:40° 34′ 13.7388′' N, 105° 4′ 57.7416′' W
**Data accessibility**	The mass spectrometry proteomics data have been deposited to the ProteomeXchange Consortium via the PRIDE public repository with the dataset identifier PXD017535: https://www.ebi.ac.uk/pride/archive/projects/PXD017535
**Related research article**	[1] C. Zhai, B.A. Djimsa, J.E. Prenni, D.R. Woerner, K.E. Belk, M.N. Nair, Tandem mass tag labeling to characterize muscle-specific proteome changes in beef during early postmortem period, J. Proteomics. (2020) 103,794.

**Value of the Data**

Protein identification and relative quantification via LC-MS/MS and TMT labeling allowed for enhanced characterization of bovine *longissimus lumborum* and *psoas major* muscles postmortem protein profiles.
Functional characterization of identified proteins shed light on the of biological pathways and cellular processes at play in early postmortem muscle.
A comparative analysis of proteome profiles from multiple timepoints revealed dynamic proteome changes in early postmortem muscle and identified candidate protein biomarkers of meat quality.

## 1 Data description

The dataset, deposited to the ProteomeXchange Consortium via the PRIDE public repository without restriction (https://www.ebi.ac.uk/pride/archive/projects/PXD017535), reports raw LC-MS/MS data examining proteins isolated from bovine *longissimus lumborum* (LL) and *psoas major* (PM) biopsy samples at three postmortem time points. Sample collection information and identification numbers are summarized in Table S1. For TMT labeling, samples were randomly assigned a TMT label and then pooled into sets of ten, as indicated in Table S2. The high pH, reverse phase fractionation gradient is shown in Table S3. After protein identification using Mascot, Scaffold software was used for post-processing including the normalization of quantitative data. A list of identified proteins with log2-transformed, normalized abundance is presented in Table S4. Statistical analysis was performed with R 3.4.3 using the limma package, and the output of the statistical analysis for each of the 9 comparisons of interest, including protein accession number, raw *p*-value, adjusted *p*-value, and fold change, are shown in Table S5. Proteins meeting the statistical standard (adjusted *p*-value < 0.05) and fold change threshold (1.5) were further characterized by enrichment analysis (*p*-value < 0.05) of Gene Ontologies (GO) for biological process and molecular function, as presented in Table S6 and Table S7, respectively.

## 2 Experimental design, materials, and methods

### 2.1 Experimental design

Bovine *longissimus lumborum* (LL) and *psoas major* (PM) muscles biopsy samples from four carcasses (*n* = 4) were obtained from a commercial packing plant at the following time intervals points postharvest: (1) 45 min, (2) 12 h, and (3) 36 h post exsanguination, resulting in a collection of a total of 24 samples (2 muscles of 4 animals at 3 different postmortem time points), as summarized in Table S1. The muscle samples were collected using a biopsy punch and were frozen immediately in liquid nitrogen until proteomic analyses. We hypothesized that proteome changes during the early postmortem period would be muscle-specific, and differential proteins would be identified between two muscle types at a given time point (PM 45 min vs. LL 45 min; PM 12 h vs. LL 12 h; and PM 36 h vs. LL 36 h) and at different time points within the same muscle (PM 45 min vs. PM 12 h; PM 45 min vs. PM 36 h; PM 12 h vs. PM 36 h; LL 45 min vs. LL 12 h; LL 45 min vs. LL 36 h; and LL 12 h vs. LL 36 h). False discovery rate and multiple testing were performed by Hochberg multiple testing adjustment at *P* < 0.05. The threshold of abundance ratio for differential protein identification was set as 1.5.

## 3 Material and methods

### 3.1 Meat sample preparation, protein extraction and quantification

Around 200 mg of muscle per sample was provided in 5 mL tubes appropriate for use in the Bullet Blender 5 Storm (Next Advance). An approximately equal volume of 3.2 mm stainless steel beads (6 beads) and 500μl lysis buffer (2.5% SDS, 1X HALT protease inhibitor (Thermo Fisher Scientific; Waltham, MA), 75 mM triethyl ammonium bicarbonate; TEAB) were added to each sample. Samples were then subjected to bead beating (speed 10 for 3 min, followed by speed 12 for 3 min). Two hundred fifty μl additional lysis buffer was added to each sample before incubation at 100 °C for 20 min and cooling on ice. Samples were then centrifuged at 5000 x g for 5 min to pellet intact cells and debris. Small aliquots were diluted to fifty times and measured for total protein content using the Pierce BCA Protein Assay Kit (Thermo Fisher Scientific). Protein reduction-alkylation and digestion were performed according to the TMT 10-plex kit instructions (Thermo Scientific).

### 3.2 Peptide labeling & cleanup

After digestion, peptide concentration was measured on a NanoDrop (Thermo Fisher Scientific) using absorbance at 205 mn and an extinction coefficient of 31 [2]. The peptide from each sample was aliquoted and raised to 100 μl using TEAB. An equal amount (66.31 μg) of the peptide from each sample was combined to make a MixQC pool. This MixQC was then separated into three aliquots and treated as a sample within each TMT set. All other samples were randomized into 3 TMT sets and assigned a TMT label (see Table S2). All TMT label reagents were equilibrated to room temperature before reconstitution in 41 μl LC-MS acetonitrile and vortexing over 5 min. 41 μl of assigned TMT label was added to each sample and incubated at room temperature for 1 hour. Label quenching was achieved by the addition of hydroxylamine (0.37% final) and 15 min incubation. TopTips (TT1000C18, PolyLC; Columbia, MD) centrifuged at 3000 rpm were used to clean up pooled, multiplexed peptides. Fifty percent Acetonitrile (ACN) was used for activation; 5% ACN, 0.5% trifluoroacetic acid (TFA) were used for equilibration and wash; 70% ACN, 0.1% formic acid (FA) were used for elution. Eluates were dried in a Savant speedvac and then reconstituted in 66 μl 5% ACN, 0.1% FA.

### 3.3 Peptide fractionation

Ten μl from each labeled sample (total 90 μg peptide from each TMT set) was subjected to high pH fractionation through a Waters Acquity UPLC BEH C18, 1.7 μm column (held at 45 °C) using a Waters H-class UPLC. Mobile phase A contained 10 mM ammonium formate, pH 10, while B consisted of 90% ACN, 10% 10 mM ammonium formate, pH10. Fractionation occurred at 100 μl/minute using the 30 min gradient shown in Table S3. A total of 48 fractions were collected in a linear fashion into four rows of a 96 well plate during peptide elution. Fractions originating from various portions of the gradient were pooled into 12 final fractions (by pooling all samples in column 1, all samples in column 2, etc.). Pooled fractions were dried in the Savant speedvac, then reconstituted in 12 μl of 5% ACN, 0.1% FA, and quantified as described above using the NanoDrop.

### 3.4 Mass spectrometry analysis

The 12 fractions from each TMT set were block randomized and injected in a randomized set order. 0.6 μg peptides were purified and concentrated on an on-line enrichment column (Waters Symmetry Trap C18 100 Å, 5 μm, 180 μm ID x 20 mm column). Chromatographic separation was conducted at a flow rate of 350 nanoliters/min on a reverse phase nanospray column (Waters, Peptide BEH C18; 1.7 μm, 75 μm ID x 150 mm column, 45 °C) using a 55-minute linear gradient from 5%−40% buffer B (100% ACN, 0.1% formic acid) followed by 40–85% buffer B over 7 min. Peptides were eluted into the mass spectrometer (Orbitrap Velos Pro, Thermo Fisher Scientific) equipped with a Nanospray Flex ion source (Thermo Scientific). Precursors were measured within a *m/z* range of 400–1500 under positive mode ionization. Fragmentation of precursors occurred using a dynamic exclusion limit of 1 MS/MS spectra of a given *m/z* value for 30 s (exclusion duration of 120 s). FT profile mode (resolution of 30,000) was used for both MS and MS/MS detection. HCD with normalized collision energy set to 35% was used for fragmentation. Xcalibur 3.0 software (Thermo Scientific) was used to generate the compound lists of the resulting spectra with a S/N threshold of 1.5 and 1 scan/group.

### 3.5 Data processing

ProteoWizard MsConvert (version 3.0) was used for extraction, charge state deconvolution and deisotope of tandem mass spectra. Spectra from all samples were searched using Mascot (Matrix Science, London, UK; version 2.6.0) against a *Bos taurus* reference proteome downloaded on February 5, 2018, from Uniprot (UP000009136) and reverse concatenated (48,676 total entries). Mascot search parameters assumed tryptic peptides and allowed a parent ion tolerance of 20 ppm and fragment ion mass tolerance of 0.020 Da. Variable modifications included oxidation of methionine, carbamidomethyl of cysteine and TMT6plex of lysine and the N-terminus. Results from each TMT set were subjected to MuDPIT, imported and combined by the probabilistic protein identification algorithms [3] in the Scaffold software (version Scaffold_4.8.4, Proteome Software Inc., Portland, OR) [4]. Peptide probability thresholds were set such that a peptide FDR of 0.04% was achieved based on hits to the reverse database [5]. Identifications of proteins were accepted if they achieved greater than 99.0% probability and contained at least 2 unique peptides. Protein probabilities were assigned by the Protein Prophet algorithm [6]. Proteins containing similar peptides and that were not differentiated based on MS/MS analysis alone were grouped to satisfy the principles of parsimony. Values supplied by Thermo Fisher Scientific (Lot SH255638) were used to correct channels according to the algorithm in [7]. Normalization was performed across samples based on intensities [8]. Medians were used for averaging. Reporter ion intensities were log-transformed and weighted by an adaptive intensity weighting algorithm. Peptide spectra assigned to multiple proteins or those missing a reference value were removed from the analysis. The list of identified proteins with log2 transformed and normalized abundance was presented in Table S4.

### 3.6 Statistical analysis

Statistical analysis was performed with R 3.4.3 using the limma package [9]. Any identified proteins that had missing values for 12 or more samples (more than half the observations) were removed. Pairwise comparisons between time points and muscles were performed by a moderated *t*-test. Benjamini–Hochberg multiple testing adjustment was used to control the false discovery rate and control for multiple testing [10] at *p* < 0.05. The output of the statistical analysis for each of the 9 comparisons of interest (PM 45 min vs. LL 45 min; PM 12 h vs. LL 12 h; PM 36 h vs. LL 36 h; PM 45 min vs. PM 12 h; PM 45 min vs. PM 36 h; PM 12 h vs. PM 36 h; LL 45 min vs. LL 12 h; LL 45 min vs. LL 36 h, and LL 12 h vs. LL 36 h) was shown in Table S5. The minimal fold change of abundance ratio for differential protein identification was set as 1.5.

### 3.7 Functional enrichment analysis

No differentially abundant proteins (*p* > 0.05) were detected within the same muscle during different time points postmortem, so only the list of the differentially abundant proteins (*p* < 0.05) across muscle type and postmortem time period (PM 45 min vs. LL 45 min; PM 12 h vs. LL 12 h; and PM 36 h vs. LL 36 h) was further characterized by enrichment analysis of Gene Ontologies (GO) terms by STRING 11 [11]. The output of enrichment of GO terms (*p* < 0.05) for biological process and molecular function are shown in Table S6 and Table S7, respectively.

## Declaration of Competing Interest

The authors declare that they have no known competing financial interests or personal relationships which have, or could be perceived to have, influenced the work reported in this article.

## Appendix Supplementary materials

Image, application 1
